# The long noncoding RNA *Six3OS *acts in *trans *to regulate retinal development by modulating Six3 activity

**DOI:** 10.1186/1749-8104-6-32

**Published:** 2011-09-21

**Authors:** Nicole A Rapicavoli, Erin M Poth, Heng Zhu, Seth Blackshaw

**Affiliations:** 1 Department of Neuroscience, Neurology and Ophthalmology, Center for High-Throughput Biology and Institute for Cell Engineering, Johns Hopkins University School of Medicine, 733 N. Broadway Avenue, Baltimore, MD 21287, USA; 2 Howard Hughes Medical Institute and Program in Epithelial Biology, Department of Dermatology, Stanford University School of Medicine, Stanford, CA 94305, USA; 3 Department of Pharmacology and Center for High-Throughput Biology, Johns Hopkins University School of Medicine, 733 N. Broadway Avenue, Baltimore, MD 21287, USA

## Abstract

**Background:**

Thousands of different long non-coding RNAs are expressed during embryonic development, but the function of these molecules remains largely unexplored.

**Results:**

Here we characterize the expression and function of *Six3OS*, a long non-coding RNA that is transcribed from the distal promoter region of the gene encoding the homeodomain transcription factor Six3. Overexpression and knockdown analysis of *Six3OS *reveals that it plays an essential role in regulating retinal cell specification. We further observe that *Six3OS *regulates Six3 activity in developing retina, but does not do so by modulating Six3 expression. Finally, we show that *Six3OS *binds directly to Ezh2 and Eya family members, indicating that *Six3OS *can act as a molecular scaffold to recruit histone modification enzymes to Six3 target genes.

**Conclusions:**

Our findings demonstrate a novel mechanism by which promoter-associated long non-coding RNAs can modulate the activity of their associated protein coding genes, and highlight the importance of this diverse class of molecules in the control of neural development.

## Background

It has recently become clear that long non-coding RNAs (lncRNAs) comprise a large fraction of the mammalian transcriptome [1]. Much effort has been focused on functional analysis of lncRNAs that are processed into short fragments, such as microRNAs, that regulate expression of protein coding genes via homologous base pairing. However, several thousand mammalian lncRNAs have been identified that span multiple kilobases in length, and in some cases show extensive conservation at the nucleotide level [2-4].

To date, only a small number of lncRNAs have been functionally characterized, although this list is growing rapidly. Some lncRNAs act via antisense base pairing to block gene expression [5-7], but many show no clear sequence overlap with the mRNAs of protein coding genes. Several of these lncRNAs are known instead to regulate mRNA transcription, acting in *cis *to regulate heterochromatin formation at nearby genomic loci. The *Xist*/*Tsix *transcripts mediate X-inactivation in placental mammals [8], and *Kcnq1ot *is important for silencing of the Kcnq locus resulting from parental imprinting [9]. Other lncRNAs regulate transcription of genes that are located great distances away from their own genomic loci. One notable example of such a *trans*-acting lncRNA is *HOTAIR*, which is transcribed from within specific Hox gene clusters, but which regulates the expression of Hox genes located on different chromosomes [10,11]. *HOTAIR*, *Kcnq1ot *and *Xist *all mediate their effects by interacting with the Polycomb-repressive complex 2 (PRC2) component Ezh2 (enhancer of zeste homolog 2 (*Drosophila*)) and modulating histone methylation [9,11,12]. Finally, a small number of lncRNAs also directly interact with transcription factors, and potentially function as transcriptional coregulators [13-15]. Although the emerging picture suggests that lncRNAs may play an important and widespread role in regulating mammalian gene expression, a central and still unresolved question is how lncRNAs act in *trans *to regulate expression of specific target genes without the use of homologous base paring.

A complex assortment of lncRNAs is expressed in the developing and mature mammalian central nervous system, with the cellular expression patterns of nearly 1, 000 different lncRNAs having been previously described [16-18]. Many show highly specific expression in specific brain regions and neuronal subtypes and it has been speculated that these lncRNAs may play a critical role in generating and maintaining the great cellular complexity found in the central nervous system [19,20]. Although a limited number of intergenic lncRNAs have been found to regulate neural development, their mode of action remains obscure [21,22]. Mechanistic insight into the function of one brain-expressed lncRNAs has come from analysis of *Evf-2*, a lncRNA co-transcribed with the homeodomain factor Dlx6. *Evf-2 *modulates transcription of *Dlx6 *by recruiting DLX2 and MECP2 to the ultraconserved *ei *enhancer element that is also transcribed as part of *Evf-2 *itself. The transcribed domain containing the *ei *sequence is essential for *Evf-2 *to activate expression of *Dlx6*, which has raised the possibility that *Evf-2 *might regulate *Dlx6 *transcription at least in part through the formation of a RNA-DNA hybrid; this hybrid may in turn facilitate binding of the *ei *sequence by DLX2 and MECP2 [14,15].

Recent studies have also begun to address the function of long non-coding opposite-strand transcripts (lncOSTs), which are divergently co-transcribed with a broad range of neuronally expressed genes. Over one-third of brain-expressed homeodomain genes possess an associated lncOST, which typically spans the promoter, but not the transcribed region, of the protein coding gene in question [23,24]. Since short promoter-associated ncRNAs can regulate expression of nearby protein coding genes [25-27], this has raised the possibility that these lncOSTs might also act in *cis *to selectively regulate the expression of their associated protein coding gene. However, although lncOSTs comprise a substantial fraction of all brain-expressed lncRNA species, their function has yet to be directly investigated.

In this study, we characterize the molecular function and mechanism of the lncOST *Six3OS*. *Six3OS *is co-expressed with the homeodomain factor Six3, a homologue of the *Drosophila sine oculis *gene [28,29]. Like *sine oculis*, *Six3 *plays a critical role in mammalian eye development, regulating both early eye formation and cell specification in the postnatal retina [30,31]. Both *Six3 *and *Six3OS *are strongly and selectively expressed in the developing mouse retina and hypothalamus [23,32,33]. We use both *in vivo *overexpression and short hairpin RNA (shRNA)-mediated knockdown analysis to analyze whether gain or loss of function of *Six3OS *results in altered differentiation of specific retinal cell subtypes. We also examine whether *Six3OS *acts cooperatively with Six3 to regulate retinal differentiation, but find that *Six3OS *does not regulate *Six3 *expression levels. Finally, we provide evidence that *Six3OS *can directly bind both to known transcriptional coregulators of *Six3 *and to histone modification enzymes, thereby functioning as an RNA-based transcriptional scaffold. These results demonstrate the mechanism by which this diverse class of molecules regulates cell specification during development.

## Results

### Genomic organization of *Six3 *and *Six3OS*

**Figure 1:**
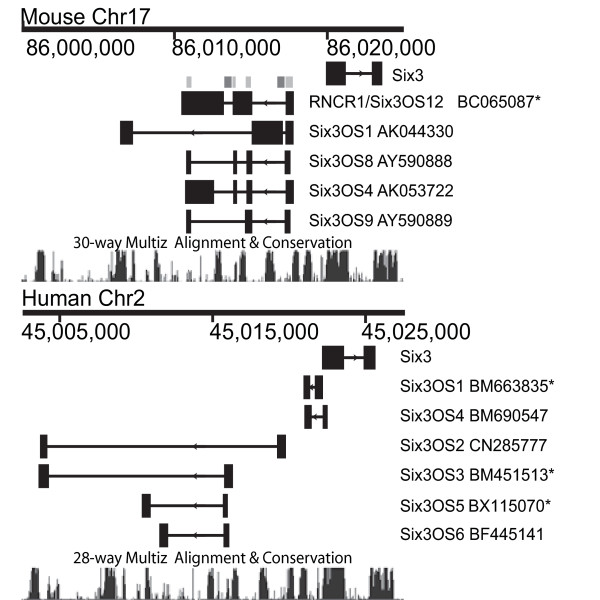
**Genomic structure and evolutionary conservation of *Six3 *and *Six3OS***. Schematic drawing showing conservation of human and mouse *Six3 *and *Six3OS *genomic structure. Light grey bars show where the predominant *Six3OS *mouse and human forms overlap. Dark grey bars indicate where mouse *Six3OS *is adjacent to conserved regions. Conservation is plotted using the PhastCons program [47]. An asterisk indicates the cRNA probes that were hybridized to the protein microarray in Figure 6.

To determine which regions of *Six3OS *to target for functional analysis, we first examined the genomic organization and evolutionary conservation of this lncOST using publicly available cDNA and genomic sequence. As previously reported, we found that *Six3OS *is alternatively spliced in both mouse and human [23], although *Six3OS*-like transcripts were not identified in any other vertebrate species examined (data not shown). To determine which sequences to target for overexpression and knockdown, we aligned these sequences and observed that the putative full-length mouse cDNA BC065087 contains the exon sequences shared by all alternative splice forms of *Six3OS *(Figure 1, light grey bars). Two of these exons are adjacent to alternatively spliced exons of the human *Six3OS *orthologue, which lie within intronic regions of the mouse transcript (Figure 1, dark grey bars). This cDNA corresponds to the most abundant isoform in neonatal retina as measured by serial analysis of gene expression (SAGE) tag abundance [32], and also matches the 4.5-kb isoform of *Six3OS*, previously reported to be the most abundant isoform expressed in embryonic brain [33]. We therefore selected this cDNA for analysis by overexpression.

### Cellular expression pattern of *Six3OS*

**Figure 2:**
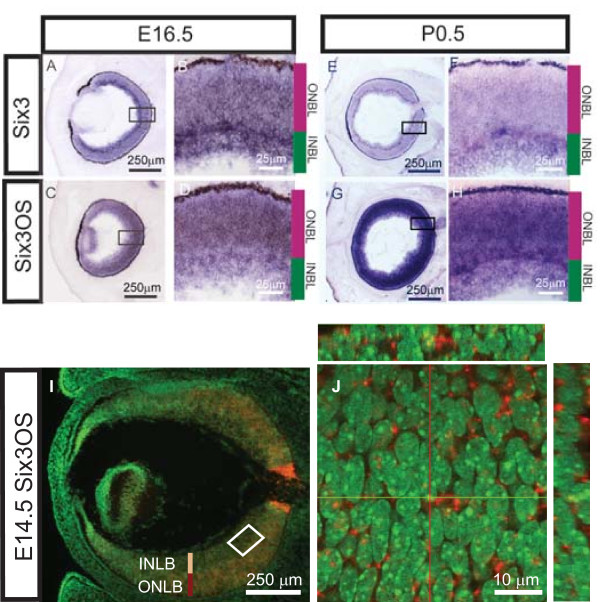
**Expression and subcellular localization of *Six3OS *and *Six3 *in the developing retina**. **(A-H) **Analysis of adjacent sections using chromogenic ISH for *Six3OS *and *Six3 *indicates substantial overlap of *Six3OS *and *Six3 *expression at E16.5 (A-D) and P0.5 (E-H). **(I, J) **Section fluorescent *in situ *hybridization (FISH) showing *Six3OS *expression. At E14.5, *Six3OS *is localized in both the nucleus (crosshairs) and the cytoplasm (I).

To determine if *Six3 *and *Six3OS *are co-expressed in retinal progenitors, as had been previously suggested [23,32,33], we performed chromogenic *in situ *hybridization (ISH) on sections of embryonic day (E)16.5 and postnatal day (P)0.5 retina (Figure 2A-H). We observed that *Six3 *and *Six3OS *are co-expressed in both retinal progenitor cells and newly post-mitotic cells of the inner neuroblastic layer. Additionally, *Six3OS *is expressed in the developing lens (Figure 2C), while *Six3 *expression is concentrated at the interface of the inner and outer neuroblastic layers (Figure 2B) at E16.5. At P0.5, *Six3OS *expression appears to be more concentrated in the outer neuroblastic layer when compared to Six3, which is primarily found in the inner neuroblastic layer (Figure 2H). The differences at the interface between these layers are more difficult to discern at P0.5, due to the increased expression of *Six3OS *in the outer neuroblastic layer (Figure 2E-H). To determine the subcellular expression pattern of *Six3OS *during development, we performed fluorescent ISH (FISH) with a probe for *Six3OS *at E14.5, at which point prominent expression in progenitor cells of the outer neuroblastic layer is seen (Figure 2I). Interestingly, though most *Six3OS *RNA is localized in the cytoplasm of retinal progenitors at this stage, some *Six3OS *RNA is also associated with nuclear DNA (Figure 2I, J). This subcellular distribution has also been noted for the lncRNA *Evf-2 *[14]. Using FISH in conjunction with immunostaining for the cytoplasmic ribosomal protein S6, we observed that the *Six3OS *transcript is also found in the nucleus and cytoplasm when expressed in transfected HeLa cells (Additional file 1).

### Overexpression and knockdown of *Six3 *and *Six3OS*

Since *Six3OS *was coexpressed with *Six3 *in retinal progenitors, we hypothesized that *Six3OS *might regulate the expression and/or activity of Six3 in developing retina. To determine if this was indeed the case, we employed *in vivo *electroporation of neonatal (P0.5) mice to determine whether overexpression and knockdown of *Six3OS *phenocopied the effects of overexpression and knockdown of *Six3*. For overexpression of *Six3OS*, we used full-length cDNAs corresponding to BC065087 (Figure 1) cloned into the pCAG plasmid [34]. Upon transfection into HeLa cells, robust *Six3OS *expression was confirmed by FISH (Additional file 1). To visualize electroporated cells, all constructs were co-electroporated with plasmids expressing green fluorescent protein (GFP) from the same CAG promoter.

**Figure 3:**
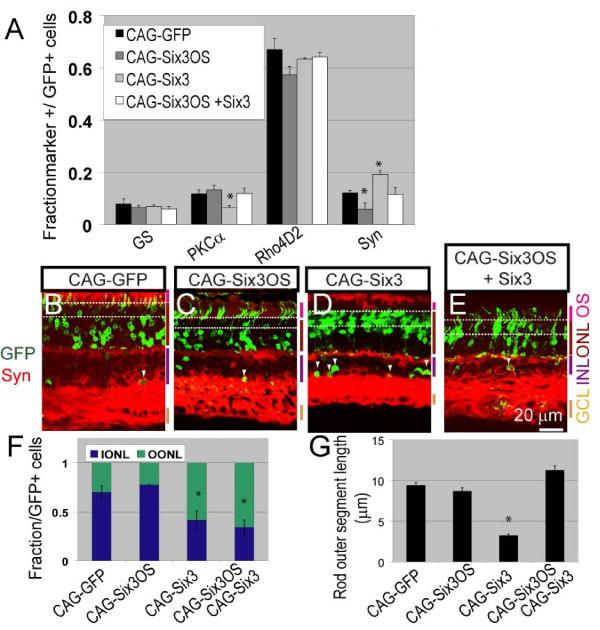
**Overexpression of *Six3OS *in developing retina inhibits changes in cell fate and photoreceptor morphology observed following *Six3 *overexpression**. **(A) **Electroporation of CAG-*Six3 *led to an increase in the fraction of GFP-positive cells expressing the amacrine cell marker syntaxin and a decrease in cells expressing the rod bipolar marker PKCα, CAG-*Six3OS *led to a decrease in syntaxin-positive cells, and co-expression led to a cell composition that was indistinguishable from CAG-GFP controls. *P < 0.05. **(B-E) **Section immunohistochemistry of retinas electroporated with CAG-GFP, CAG-*Six3OS*, CAG-*Six3 *or both CAG-*Six3OS *and CAG-*Six3*. White dashed lines define the outer third of the outer nuclear layer (OONL). Syntaxin (red) is co-immunostained with GFP (green). (C) No obvious difference is observed in either amacrine cell number or morphology (white arrowheads) in retinas electroporated with CAG-*Six3OS *relative to CAG-GFP controls or in the fraction of the cells in the OONL. (D) An increase in amacrine cell number and in the number of cells in the OONL and a decrease in outer segment length is observed in the case of *Six3*. (E) An increase in the number of cells in the OONL is observed, but no difference in amacrine cell number or outer segment length is observed from controls following co-electroporation of CAG-*Six3 *and CAG-*Six3OS*. GCL, ganglion cell layer; INL, inner nuclear layer; ONL, outer nuclear layer; OS, outer segment.. **(F) **Laminar position of cells within the OONL. Electroporation of CAG-*Six3 *leads to a shift of rod photoreceptor cell bodies to the OONL, and this effect is not affected by co-electroporation of CAG-*Six3OS *(white dashed lines in B-E). **(G) **Rod photoreceptors electroporated with CAG-*Six3 *show substantially shorter outer segments and this effect is reversed by co-electroporation with CAG-*Six3OS*. Cell type specific markers used: rhodopsin (Rho4D2), rod photoreceptors; glutamine synthetase (GS), Muller glia; protein kinase C alpha (PKCα), rod bipolar cells; syntaxin (Syn), amacrine cells.

We observed that overexpression of 1 μg of *Six3OS *resulted in no significant change in any major retinal cell type, and section immunohistochemistry revealed that electroporated cells showed grossly normal morphology (Figure 3B, C, white arrowheads). However, when dissociated cell preparations were examined, a significant decrease (*P *< 0.05) in the fraction of GFP-positive cells expressing amacrine cell-specific marker syntaxin was observed relative to cells electroporated with the GFP control vector at P21 (Figure 3A). Since *Six3OS *overexpression did not alter the fraction of GFP-positive cells with amacrine-like morphology and laminar position, we conclude that *Six3OS *does not inhibit amacrine cell specification. The reduction in syntaxin expression seen following CAG-*Six3OS *electroporation thus reflects a quantitative reduction in syntaxin levels or possibly reflects reduced adhesion of *Six3OS*-expressing amacrine cells following dissociation for immunocytochemistry.

We next tested the effects of overexpression of pCAG-*Six3 *using *in vivo *electroporation. Upon electroporation, expression of Six3 was confirmed by section immunohistochemistry. Cell fate specification in the postnatal retina is highly sensitive to Six3 dosage, and both gain and loss of function of Six3 have been reported to result in similar defects in bipolar cell and photoreceptor development [30]. We observed that electroporation of 1 μg of pCAG-*Six3 *led to a reduction in the number of rod bipolar cells, and detected an increase in the fraction of GFP-positive amacrine cells by P21 (*P *< 0.05; Figure 3A, D). The length of the outer segments of GFP-positive rod photoreceptors was also decreased (*P *< 0.05; Figure 3D, G). Finally, we observed that the cell bodies of the *Six3 *electroporated photoreceptor cells were primarily located in the outer third of the outer nuclear layer (*P *< 0.05; Figure 3D, white dashed line, and 3F), in contrast to photoreceptors electroporated with control vector, which were distributed throughout the outer nuclear layer.

We then analyzed the effects of loss of function of *Six3OS *and *Six3*. To this end, we first tested shRNAs for their ability to reduce expression of endogenous *Six3 *and *Six3OS *expression by *in vivo *electroporation of P0.5 retina. Reduction in expression of the target gene was determined by analyzing expression of either Six3 protein or *Six3OS *RNA dissociated GFP-positive cells. For both *Six3 *and *Six3OS*, individual shRNA constructs were identified that resulted in a substantial reduction in the average fluorescence intensity in either Six3 protein or *Six3OS *RNA in GFP-positive cells (Additional file 2).

**Figure 4:**
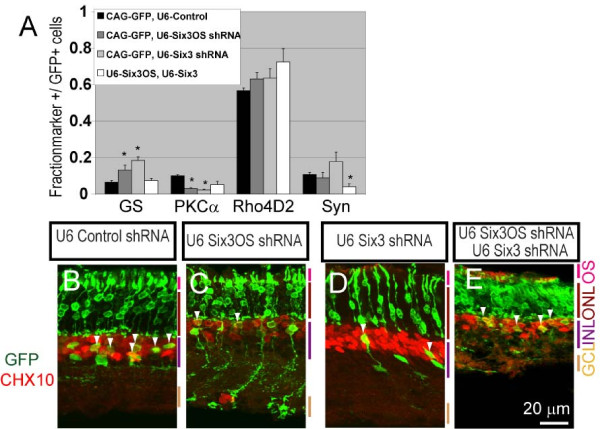
**shRNA-mediated knockdown of *Six3 *and *Six3OS *in the developing retina alters bipolar cell and Muller glial development**. **(A) ***Six3 *knockdown and *Six3OS *knockdown both lead to an increase in Muller glia and a decrease in rod bipolar cells as measured by immunostaining of dissociated electroporated retinal cells. Simultaneous knockdown of both *Six3 *and *Six3OS *resulted in a normal number of rod bipolar and Muller glia, but a decrease in syntaxin (Syn)-positive amacrine cells was observed. *P < 0.05. GS, glutamine synthetase. **(B-E) **Section immunohistochemistry demonstrates a decrease in GFP-positive bipolar cells following knockdown of either *Six3OS *or *Six3*, but not both in combination (white arrowheads). Chx10 (ceh-10 homeodomain containing homolog (*C. elegans*)) was used as pan-bipolar cell marker. GCL, ganglion cell layer; INL, inner nuclear layer; ONL, outer nuclear layer; OS, outer segment.

A significant decrease in the fraction of protein kinase C α (PKCα) positive rod bipolar cells was observed at P21 following electroporation of either 1 μg of *Six3 *shRNA construct or 1 μg of *Six3OS *shRNA (*P *< 0.05; Figure 4A), reminiscent of effects of retroviral overexpression of a dominant-negative mutant form of *Six3 *[30]. The fraction of glutamine synthetase-positive Muller glia was also increased (*P *< 0.05; Figure 4A). Section immunohistochemistry confirmed an increase in glutamine synthetase-positive cells with Muller glia-like morphology, at the expense of cells expressing the bipolar cell marker CHX10 and demonstrating bipolar-like morphology (Figure 4B-D, white arrowheads). To further confirm that knockdown experiments did indeed result in a selective loss of function of *Six3OS*, we also inhibited *Six3OS *function by overexpression of *Six3OS*-IRES-GFP. Fusion of IRES-GFP to lncRNAs results in a mislocalization of lncRNAs to the ribosome, and can produce dominant negative phenotypes when overexpressed [22]. When this is performed, we observed a reduction in rod bipolar markers and an increase in Muller markers, phenocopying the effects of *Six3OS *knockdown (data not shown).

### Simultaneous overexpression and knockdown of *Six3 *and *Six3OS *demonstrates non-additive effects on retinal differentiation

Having observed that loss of function of *Six3 *and *Six3OS *resulted in similar phenotypes in developing retina, we next investigated whether these two genes acted cooperatively or independently to regulate retinal differentiation. We first used *in vivo *electroporation to overexpress 1 μg of both the *Six3 *and *Six3OS *constructs simultaneously in P0.5 retina. We observed that co-expression of *Six3 *and *Six3OS *resulted in a normal cell fate phenotype (Figure 3A). The length of GFP-positive rod photoreceptor outer segments was indistinguishable from controls (Figure 3B, E, G). However, the cell bodies of the electroporated photoreceptor cells were primarily located in the outer third of the outer nuclear layer (*P *< 0.05; Figure 3B, E, white dashed line, and 3F). We thus concluded that overexpression of *Six3OS *was largely able to reverse the cellular phenotypes observed following overexpression of *Six3*.

As a follow up to these experiments, we simultaneously reduced expression of *Six3 *and *Six3OS *by co-electroporating 1 μg of the *Six3 *shRNA construct and 1 μg of *Six3OS *shRNA construct. We observed that simultaneous knockdown of both *Six3 *and *Six3OS *resulted in a normal number of bipolar cells and Muller glia. However, we observed a significant decrease in the fraction of GFP-positive amacrine cells, which was not observed following electroporation of either the *Six3 *or *Six3OS *shRNA alone (*P *< 0.05; Figure 4A, E). These data show a non-additive effect of simultaneous loss of function of both *Six3 *and *Six3OS*, and show that these genes functionally interact to regulate retinal differentiation.

**Figure 5:**
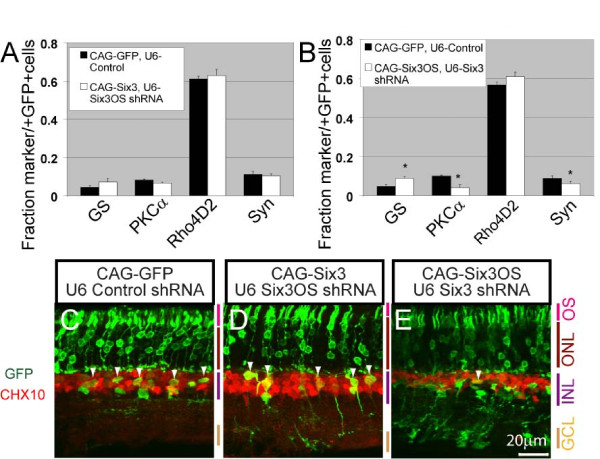
***Six3 *overexpression rescues the *Six3OS *knockdown phenotype whereas *Six3OS *overexpression is not sufficient to rescue the loss of *Six3***. **(A) ***Six3OS *knockdown combined with *Six3 *overexpression led to a cell type distribution indistinguishable from cells electroporated with control constructs when tested by immunostaining of dissociated electroporated retinal cells. **(B) ***Six3 *knockdown combined with *Six3OS *overexpression led to an increase in Muller glia and a decrease in rod bipolar cells and a decrease in syntaxin (Syn)-positive amacrine cells. GS, glutamine synthetase. **(C-E) **Section immunohistochemistry of retinas confirm a distribution of GFP-positive cells that is indistinguishable from controls when *Six3OS *knockdown is combined with *Six3 *overexpression, while *Six3 *knockdown and *Six3OS *overexpression led to a decrease in bipolar cells (F, white arrowheads). GCL, ganglion cell layer; INL, inner nuclear layer; ONL, outer nuclear layer; OS, outer segment.

Lastly, we directly investigated the functional relationship between *Six3 *and *Six3OS in vivo *by simultaneously overexpressing *Six3 *and knocking down *Six3OS*, and *vice versa*. When we co-electroporated 1 μg of CAG-*Six3 *with 1 μg of *Six3OS *shRNA, we found that *Six3 *overexpression fully rescued the *Six3OS *knockdown phenotype. Furthermore, loss of function of *Six3OS *fully eliminated the effects of *Six3 *overexpression. No differences in the composition of GFP-positive cells were observed when compared to controls (Figure 5A). Both the morphology and distribution of the cells within the retina were normal (Figure 5C, D; Additional file 3A), and the rod photoreceptor outer segment length was normal (Additional file 3B). We conclude that *Six3OS *is required for many of the effects on retinal differentiation that result from *Six3 *overexpression.

In contrast, when 1 μg of CAG-*Six3OS *was co-electroporated with 1 μg of *Six3 *shRNA, we observed an increase in glutamine synthetase-positive cells with Muller glia-like morphology (*P *< 0.05; Figure 5B) and a decrease in PKCα-positive and CHX10-positive bipolar cells (*P *< 0.05; Figure 5B, C, E) when compared to controls. Additionally, a decrease in cells expressing the amacrine cell-specific marker syntaxin was observed (*P *< 0.05; Figure 5B). These retinas, however, do not show any change in the number of GFP-positive cells with amacrine cell-like morphology. We therefore conclude that, as is the case when *Six3OS *alone was overexpressed, *Six3OS *overexpression in conjunction with knockdown of *Six3 *reduces syntaxin expression without otherwise affecting amacrine cell differentiation (Figure 5B, E). The phenotype seen here is the sum of the phenotypes seen following *Six3OS *overexpression and *Six3 *knockdown, and stands in contrast to the non-additive phenotype seen when *Six3OS *and *Six3 *are both simultaneously knocked down (Additional file 4). We thus conclude that the phenotype observed following *Six3OS *overexpression is not affected by *Six3 *loss of function, and that *Six3OS *may also regulate syntaxin expression through a *Six3*-independent mechanism.

### *Six3OS *does not directly regulate Six3 protein levels

Although these data suggest that *Six3OS *regulates *Six3 *function in developing retina, they do not directly address the molecular mechanism by which *Six3OS *is able to do so. A number of studies have suggested that both promoter and enhancer-associated lncRNAs can act in *cis *to regulate expression levels of nearby protein-coding genes [14,25-27,35,36]. To investigate whether *Six3OS *might be regulating Six3 function by a similar mechanism, we overexpressed and knocked down expression of *Six3OS *at P0.5 in retina, and quantified the level of Six3 protein in GFP-positive cells at P5.5. We did not observe any change in Six3 protein levels following either overexpression or knockdown of *Six3OS *(Additional file 5A, B). Furthermore, we tested whether *Six3OS *could regulate Six3-dependent regulation of a reporter construct derived from the sequence of the mouse *Six3 *promoter, which was previously shown to be negatively regulated by Six3 [30]. To determine if *Six3OS *modulates Six3-mediated auto-repression, CAG-*Six3OS *was cotransfected with the CMV-*Six3 *and the *Six3*-pro luciferase vector. As previously reported, overexpression of *Six3 *represses this reporter construct when tested in NIH3T3 cells. However, *Six3OS *overexpression had no effect on Six3-mediated auto-repression (Additional file 6). Although 14 additional human and mouse cell lines were tested, no *Six3OS*-dependent effects on Six3-mediated autorepression could be detected (data not shown). We conclude that transcription of *Six3 *mRNA is not regulated by *Six3OS*. Instead *Six3OS *RNA likely modulates the ability of Six3 protein to activate or repress expression of its target genes in developing retinal cells.

### Ezh2 and Eya1/3/4 directly bind *Six3OS*

To identify proteins that directly bind *Six3OS*, Cy5-labeled *Six3OS *RNA was used to probe human protein microarrays [37]. Mouse *Six3OS *and three human splice variants of *Six3OS *were transcribed *in vitro *and labeled with Cy5, and antisense cRNAs for these same transcripts were hybridized in parallel as negative controls. A total of five proteins that specifically and selectively interact with both the mouse and human forms of *Six3OS *were thus identified. We found that both human and mouse *Six3OS *selectively interact with Eya1, encoded by a homologue of the *eyes absent *gene of *Drosophila*. In addition, *Six3OS *directly binds to the chromatin remodeling enzyme subunits Smarce1 and Ezh2, as well as Eno1 and Ppp5c (Additional file 7). Members of the Eya protein family have been previously shown to interact with Six family proteins [38], but Eya1 is not expressed in the mouse neuroretina [32]. Therefore, we also performed RNA immunoprecipitation (RIP) experiments on Eya3 and Eya4, which are both robustly expressed in embryonic retina.

**Figure 6:**
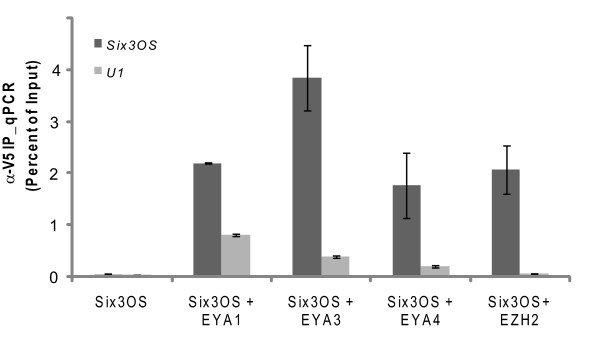
***Six3OS*-protein interactions detected via protein microarray**. αV5-RIP specifically retrieves Eya1-V5, Eya3-V5, Eya4-V5 and Ezh2-V5 bound to *Six3OS *RNA but not U1 small nuclear RNA. Mean ± standard deviation, n = 3, relative to input is shown.

To test whether Ezh2 and Eya family members interact with mouse *Six3OS in vivo*, co-immunoprecipitation experiments were performed in HEK 293T cells overexpressing V5-tagged Eya1, Eya3, Eya4 and Ezh2. Expression and immunoprecipitation of V5-tagged protein was verified by immunoblot (Additional file 8). We confirmed that mouse *Six3OS *RNA selectively interacts with each of these proteins using RIP (Figure 6), and conclude that these proteins interact with *Six3OS *in transfected HEK 293T cells. To confirm that *Six3OS *interacts with Eya family members and Ezh2 specifically, we examined whether *Six3OS *interacts directly with Six3 protein by RIP and found that *Six3OS *and Six3 do not interact in transfected HEK 293T cells (data not shown). This suggests that *Six3OS *regulates Six3 function by facilitating interaction between Eya proteins and chromatin-modifying enzyme complexes.

## Discussion

### *Six3OS *and Six3 modulate retinal cell specification and differentiation

Our findings demonstrate that both gain and loss of function of *Six3OS *and *Six3 *affect retinal differentiation (Additional file 4). Knockdown of *Six3OS *resulted in a decrease in the fraction of bipolar cells and an increase in Muller glia in electroporated cells. An identical phenotype was observed following knockdown of *Six3 *(Figure 4). Knockdown of either *Six3OS *or *Six3 *phenocopies the effect of retroviral overexpression of a dominant-negative form of *Six3 *in neonatal retina, where a substantial decrease in bipolar cells was also seen [30].

In contrast, overexpression of either *Six3 *or *Six3OS *alone produced very different phenotypes. *Six3OS *overexpression resulted in a reduction in syntaxin staining in electroporated amacrine cells, but did not alter the morphology or laminar position of electroporated cells. On the other hand, *Six3 *overexpression, like *Six3 *loss of function, resulted in a dramatic reduction in the number of bipolar cells (Figure 3A), and also resulted in decreased rod photoreceptor outer segment length at P21 (Figure 3D, G). These effects were previously reported following retroviral-mediated overexpression of *Six3 *[30]. Interestingly, we also observed phenotypes that were not reported in this previous study, such as an increase in amacrine cells. A number of technical differences between these two experiments may account for these differences, most notably the fact that electroporation-mediated overexpression typically results in much higher levels of construct expression in developing retina than does retroviral transduction [34].

### *Six3OS *regulates Six3 activity in developing retina, but does not regulate *Six3 *expression levels

If Six3 and *Six3OS *act independently to control retinal cell specification, we would expect to observe purely additive phenotypes when their activities are both altered. Strikingly, when both *Six3OS *and *Six3 *were overexpressed simultaneously, we no longer observed the cellular phenotypes that resulted from overexpression of *Six3 *alone. Simultaneous knockdown of both *Six3OS *and *Six3 *also showed non-additive effects. The loss of bipolar cells and increase in Muller glia, which was seen following knockdown of each gene individually, were not observed when expression of both *Six3 *and *Six3OS *was reduced. Instead, a decrease in amacrine cells was observed, which was a phenotype not observed when each individual gene was knocked down. These non-additive phenotypes demonstrate an epistatic relationship between Six3 and *Six3OS*, and imply that they functionally interact to regulate retinal differentiation.

Having demonstrated that Six3 and *Six3OS *interact to regulate retinal differentiation, we next investigated whether defects in Six3 could be rescued by overexpression of *Six3OS*, and *vice versa*. When *Six3 *is overexpressed but *Six3OS *is knocked down, none of the phenotypes normally seen following either *Six3 *overexpression alone or *Six3OS *knockdown alone were observed (Figure 5A, C-D). These data indicate that loss of function of *Six3OS *suppresses the effects of both gain and loss of function of *Six3*. However, simultaneous overexpression of *Six3OS *and knockdown of *Six3 *resulted in a purely additive phenotype that was the sum of the effects of *Six3 *knockdown and *Six3OS *overexpression. These findings suggest that *Six3OS *acts to regulate Six3 activity, since overexpression of *Six3 *can rescue the effects of *Six3OS *knockdown, but not *vice versa*. As *Six3OS *overexpression leads to a reduction in syntaxin expression even following *Six3 *knockdown, this implies that *Six3OS *may also regulate retinal differentiation through a Six3-independent mechanism, although this remains to be characterized further.

Since both promoter- and enhancer-associated ncRNAs have been previously thought to act in *cis *to selectively regulate expression of nearby protein coding genes [36,39], our finding that *Six3OS *did not regulate Six3 expression came as something of a surprise. Our finding that *Six3OS *acts in *trans *to regulate retinal differentiation was supported by several lines of evidence. First, by the finding that the activity of Six3 expressed from the CAG promoter can be modulated by overexpression or knockdown of *Six3OS *(Figure 5). Second, we have directly demonstrated that neither overexpression nor knockdown of *Six3OS *has any detectable effect on *Six3 *expression in retina, nor does *Six3OS *have an effect on Six3-dependent autorepression when measured by luciferase analysis in transfected tissue culture cells. These data imply that *Six3OS *and perhaps other lncOSTs do not regulate transcription of their associated protein coding genes, but instead act in *trans *to regulate the activity directly via protein-RNA interactions. This mechanism may not hold true for all promoter-associated lncRNAs, however, and each will have to be characterized independently to determine its mechanism of action.

### *Six3OS *selectively interacts with Eya family members and Ezh2

While these data demonstrate a functional relationship between *Six3OS *and Six3, they still leave the precise mechanism by which *Six3OS *regulates Six3 activity unresolved. Our finding that both human and mouse isoforms of *Six3OS *interact with multiple different members of the *eyes absent *protein family demonstrates how this might occur *in vivo*. The *eyes absent *(*eya*) gene and its mammalian homologues encode protein tyrosine phosphatases that function as transcriptional coregulators. *Eya *binds directly to *sine oculis*, and acts in conjunction with *sine oculis *in controlling eye field specification in *Drosophila *[28,29]. We also observe that *Six3OS *directly binds the PRC2 subunit Ezh2, and also possibly the BAF57 subunit Smarce1. Additional confirmation that *Six3OS *interacts with Ezh2 *in vivo *comes from a recent study of polycomb-associated RNAs, which found that *Six3OS *was co-precipitated with PRC2 in embryonic stem cells [40]. We propose that *Six3OS *can modulate the expression of Six3 target genes without necessarily regulating expression of *Six3 *itself by acting as a transcriptional scaffold. When Six3 directs the Six3-Eya complex to bind to specific genomic target sequences, *Six3OS *may thus act as a transcriptional scaffold, recruiting histone modifying enzyme complexes to regulate expression of Six3 target genes. Although *trans*-acting lncRNAs have previously been found to regulate gene expression through recruitment of the Ezh2-containing PRC2 histone methyltransferase complex, their action is restricted to a small subset of target genes, and the mechanism by which this occurs is unknown [11]. Our findings point towards a plausible mechanism by which this could occur. However, in the absence of RNA-IP data that clearly demonstrate interaction of *Six3OS *with both Eya proteins and Six3 in developing retina, other potential mechanisms of action of *Six3OS *remain plausible.

Several unresolved questions remain, the most obvious being which Eya subtype is the most relevant target of *Six3OS in vivo*. Eya1 is known to bind Six3 both *in vitro *and in transfected tissue culture cells [41], although the co-expression of Six3 and Eya1 is limited to the ciliary margin and lens [42]. Eya2, Eya3 and Eya4, however, are all co-expressed with Six3 and *Six3OS *in both the developing retina and in other forebrain regions [42]. Eya4 and Six3 have been demonstrated to interact in a study of holoprosencephaly, indicating that Eya4 may be the functional bridge between Six3 and *Six3OS *in the developing ventral forebrain [43]. Our findings indicate that *Six3OS *may also regulate retinal differentiation by Six3-independent mechanisms. Since Eya family members can interact with other classes of transcription factors, including Hox and Tlx family members [44], this suggests that *Six3OS *might also modulate the expression of genes regulated by these transcription factors.

Many homeodomain transcription factors that are essential for central nervous system development possess an associated lncOST, including Pax6, Rax, Vax2 and Six6 [23,45]. Each of these proteins exhibits complex and dynamic expression during development, and their expression patterns often diverge considerably from those of their associated lncOST. Although their target sequences are present in all cells, the genes that are directly regulated by each protein can vary considerably, both at different developmental stages and in different cell types. Models that explain how this context-dependent regulation of transcription factor activity occurs have typically emphasized combinatorial regulation by other proteins, or cell- and stage-specific epigenetic modifications. Our finding that lncOSTs can modulate the activity of their associated transcription factors lends an additional layer of complexity to these models, and suggests that this diverse class of molecules may play a critical function in generation of cell subtype diversity within the developing central nervous system.

## Materials and methods

### Animals

Pregnant CD-1 mice were purchased from Charles River Breeding Laboratories, Wilmington, MA, USA. Animal experiments were approved by the Johns Hopkins University Institutional Animal Care and Use Committee.

### *In vivo *electroporation

*In vivo *electroporation of mouse retina was performed as described [34]. Retinas were electroporated at P0.5. Dissociated cells and section immunohistochemistry data shown here were performed at P21. Error bars represent ± standard error of the mean for at least three independent electroporated retinas. A two-tailed Students *t*-test was performed to determine the *P*-value. Control and experimental constructs were co-electroporated with 0.2 μg of pCAG-GFP to readily visualize electroporated cells by GFP [34]. For overexpression experiments, 1 μg of pCAG-GFP was injected with either 1 μg of pCAG-*Six3 *or 1 μg of CAG-*Six3OS*. For simultaneous overexpression of *Six3 *and *Six3OS*, retinas were electroporated with 1 μg of pCAG-*Six3 *and 1 μg of CAG-*Six3OS*. For knockdown of *Six3 *and *Six3OS*, 1 μg of U6-control was injected with either 1 μg of U6-*Six3 *or 1 μg of U6-*Six3OS*. For simultaneous knockdown, 1 μg of U6-*Six3 *and 1 μg of U6-*Six3OS *were injected. For overexpression of *Six3 *in combination with knockdown of *Six3OS*, retinas were electroporated with 1 μg of pCAG-*Six3 *and 1 μg of U6-*Six3OS *were injected. Finally, for overexpression of *Six3OS *in combination with knockdown of *Six3*, 1 μg of pCAG-*Six3OS *and 1 μg of U6-*Six3 *were injected and electroporated.

### *In situ *hybridization

For cryosections, untimed E14.5 and E16.5 embryos and P0.5 neonates were dissected, fixed overnight in 4% paraformaldehyde in PBS at 4°C and cryoprotected overnight in 30% sucrose/PBS at 4°C before being embedded in OCT compound (Sakura, Torrance, CA, USA) on dry ice. Cryosections (20 μm) were cut on a cryostat. Section ISH methodology was as previously described [32] with the exception that probe BC065087 was used and a Tyramide Signal Amplification system (TSA Plus, Perkin Elmer, NEL 744, Waltham, MA, USA) combined with an antidigoxigenin-HRP antibody (1:1, 000; Roche, Indianapolis, IN, USA). for fluorescent ISH. Fluorescent ISH sections were counterstained with DAPI. Fluorescent ISH samples were photographed on a Zeiss Meta 510 confocal microscope. Chromogenic images were visualized on a Zeiss Axioskop2 microscope.

### Immunohistochemistry

For cryosections, electroporated eyes were harvested 21 days after electroporation, fixed for 1 hour in 4% paraformaldehyde in PBS at 4°C and cryoprotected overnight in 30% sucrose/PBS at 4°C before being embedded in OCT compound (Sakura) on dry ice. Cryosections (20 μm) were cut on a cryostat. Retinal cryosections were immunostained as described except that sections were post fixed in 4% paraformaldehyde for 5 minutes prior to blocking [21]. Samples were photographed on a Zeiss Meta 510 confocal microscope.

For cell marker analysis, dissociation and immunostaining were performed as described [34] except that retinas were harvested at P21. Samples were visualized and quantified on a Zeiss Axioskop2 microscope. To quantify the effects of *Six3 *knockdown, retinas were electroporated with *Six3 *shRNA, harvested at P4.5, dissociated, and immunostained as described above with anti-Six3 and detection with Alexa568 goat anti-guinea pig IgG and rabbit anti-GFP and detection with Alexa488 goat anti-rabbit. For Six3 quantification, *Six3OS *overexpression and *SixOS *shRNA electroporated retinas were harvested at P5.5, dissociated and immunostained as described above with anti-Six3 and detection with Alexa568 goat anti-guinea pig IgG and rabbit anti-GFP and detection with Alexa488 goat anti-rabbit. Samples were visualized on a Zeiss Axioskop2 microscope and signal intensity was quantified with Velocity 4.0 (Perkin Elmer) software by calculating the average signal intensity per cell and normalized to cell size.

### *In situ *hybridization and immunocytochemistry

For *Six3OS *knockdown quantification, retinas were harvested at P4.5 and dissociated as described above. FISH was performed as described using TSA Plus Cyanine3 kit (1:125; Perkin Elmer, NEL 744) followed by staining as described (with rabbit anti-GFP and detection with Alexa488 goat anti rabbit). Samples were visualized and quantified as above. For all dissociations, nuclear DNA was visualized with DAPI counterstaining. Cell counts were analyzed using the two-tailed Student's *t*-test. A minimum of three retinas were counted for each construct examined using dissociated immunocytochemistry, with 100 to 300 GFP-positive cells per retina counted for each marker tested.

HeLa cell FISH followed by immunocytochemistry was performed as previously described [22] except that 20 cells were counted.

### Luciferase reporter assays

NIH 3T3 cells were grown in DMEM supplemented with 10% FCS. Cells were transfected with Fugene6 (Roche) per the manufacturer's instructions. Cells were transfected with 500 ng of luciferase reporter construct and 50 ng of the expression plasmids for *Six3 *and *Six3OS*. Cells were harvested 2 days post-transfection. Luciferase was measured per manufacturer's instructions with the Dual-Luciferase Reporter System (Promega, E1910, Madison, WI, USA). pTK-Renilla (50 ng) was co-transfected to control for transfection efficiency.

### Synthesis of RNA probes for protein microarray

BC065087 (5 μg) was linearized with DraI, and 5 μg of BM663835, BX115070, and BM451513 were linearized with NotI. Linearized DNA was then phenol-chloroform extracted, ethanol precipitated and resuspended in 10 μl water. Probes were *in vitro *transcribed with T7 polymerase per manufacturer's instructions using the Riboprobe Combination system kit (Promega, P1405), spiking the reaction with 1 μl of 5 mM Cy5 labeled CTP (GE Healthcare, 25-8010-87, Piscataway, NJ, USA). The RNA probe was ethanol precipitated with LiCl and resuspended in 1 mM EDTA.

### Protein microarray analysis

Transcription factor/RNA binding protein chips were generated as previously described [37]. Protein chips were preblocked with Superblock Buffer (Thermo Scientific, 37516, Waltham, MA, USA) supplemented with 10 μg ml^-1 ^sperm DNA, 2 mM MgCl_2_, 2 mg ml^-1 ^BSA for 1 hour at room temperature. RNA was hybridized at 250 nM concentration in binding buffer (PBS supplemented with 2 mM MgCl_2_, 2 mg ml^-1 ^BSA) at room temperature for 1 hour. The slides were washed four times in TBST, dried and scanned by a GenePix 400B scanner. Data were analyzed with GenePix Pro 6.10 as previously described [46].

### RNA immunoprecipitation experiments

HEK 293T cells were grown in DMEM supplemented with 10% FCS. Cells were transfected with Fugene6 (Roche) per the manufacturer's instructions. Ten million cells were transfected 24 hours post-plating with 5 μg of pCAGIG-V5, pCAGIG-Six3-V5, pCAGIG Eya1-V5, pCAGIG Eya3-V5, pCAGIG Eya4-V5 or pCAGIG Ezh2-V5, and pCAG-*Six3OS*. Cells were harvested 2 days post-transfection, lysed and precipitated essentially as previously described [11], except that 5 μg anti-V5 antibody was used (1:5, 000; Invitrogen, R96025, Carlsbad, CA, USA) with 50 μl protein A-agarose (Invitrogen, 15918-014) in each immunoprecipitation reaction. For each sample, 10% of total volume was set aside after lysis for RNA extraction and 5% set aside for immunoblot analysis. After precipitation, 80% of the beads were resuspended in Trizol and 20% were resuspended in Laemmle buffer. RNA was Trizol extracted and resuspended in 50 μl of nuclease free water. RNA was then DNAse treated with Turbo DNA-free (Ambion, AM1907, Carlsbad, CA, USA) per manufacturer's instructions. RNA was quantified by quantitative RT-PCR using Brilliant II SYBR Green QRT_PCR Master Mix (Agilent, 600825, Santa Clara, CA, USA) on a Roche LightCycler480. No-RT controls were simultaneously performed to demonstrate that signal was not from DNA contamination.

Expression of V5-tagged protein was confirmed by western analysis using anti-V5 antibody (1:10, 000; Invitrogen, R96025) dilution in 5% milk in PBST, and detected with horse radish peroxidase goat anti-mouse IgG (1:10, 000; Santa Cruz, sc-2031, Santa Cruz, CA, USA) and ECL Western Blotting Detection System (GE Healthcare, RPN2132 Piscataway, NJ, USA) per the manufacturers' instructions.

Full details of all plasmids and antibodies used in this study are included in Additional File 9.

## Abbreviations

BSA: bovine serum albumin; DMEM: Dulbecco's modified Eagle's medium; E: embryonic day; FCS: fetal calf serum; FISH: fluorescent *in situ *hybridization; GFP: green fluorescent protein; ISH: *in situ *hybridization; lncOST: long non-coding opposite-strand transcript; lncRNA: long non-coding RNA; ncRNA: non-coding RNA; P: postnatal day; PBS: phosphate-buffered saline; PKC: protein kinase C; PRC2: Polycomb-repressive complex 2; RIP: RNA immunoprecipitation; shRNA: short hairpin RNA; TBST: Tris- buffered saline with Tween-20.

## Competing interests

The authors declare that they have no competing interests.

## Authors' contributions

NAR designed and performed experiments, analyzed the data and wrote the manuscript. EMP performed experiments. HZ analyzed the data. SB designed experiments, analyzed the data and wrote the manuscript. This manuscript has been seen and approved by all authors.

## Supplementary Material

Additional file 1 ***Six3OS *is localized equally in the nucleus and cytoplasm**. HeLa cells were transfected with *Six3OS *constructs and RNA location was analyzed by FISH followed by immunohistochemistry against the cytoplasmic S6 ribosomal protein. Cytoplasmic *Six3OS *RNA was identified by localization with S6 protein. The relative proportion of nuclear *Six3OS*, defined as FISH signal that did not colocalize with S6 protein, is indicated. N = 20 cells. Click here for file

Additional file 2 **Confirmation of shRNA-mediated knockdown of endogenous *Six3 *and *Six3OS *in developing retina**. (A-E) A construct encoding either control hairpin, *Six3 *targeted hairpin or *Six3OS *targeted hairpin was electroporated *in vivo*, into P0.5 retina and harvested at P4.5, and dissociated, and immunocytochemistry against Six3 and GFP, or FISH against *Six3OS *was performed followed by immunostaining for GFP. GFP-positive cells were counted to analyze the fluorescence intensity for each cell that expressed (A) Six3 or (D) *Six3OS*. At least 100 GFP-positive cells from three different electroporated retinas were counted for each construct tested. Error bars represent standard error for at least three independent retinas. (A) *P *< 0.05; (D) *P *< 0.05. (B-E) Examples of dissociated cells positive for GFP and either Six3 protein or *Six3OS *RNA. Click here for file

Additional file 3 ***Six3OS *knockdown rescues changes in retinal cell morphology and position observed following overexpression of *Six3***. (A, B) *Six3OS *knockdown combined with *Six3 *overexpression rescues the effects observed on rod photoreceptor cell body position (A) and on photoreceptor outer segment length observed with *Six3 *overexpression (B). Click here for file

Additional file 4 **Summary of overexpression and knockdown data for *Six3 *and *Six3OS***. These results demonstrate that co-expression of *Six3OS *and *Six3 *rescues the phenotypes observed with *Six3 *overexpression except that the photoreceptors are displaced in the outer third of the outer nuclear layer. Simultaneous knockdown of *Six3OS *and *Six3 *results in a novel phenotype, fewer amacrine cells. Additionally, *Six3OS *overexpression rescues the phenotype of knockdown of *Six3*. However, expression of *Six3 *combined with knockdown of *Six3OS *results in an additive phenotype. Click here for file

Additional file 5 **Cellular levels of Six3 protein are not affected by overexpression or knockdown of *Six3OS***. **(A) **Overexpression of *Six3OS *at P0.5 does not affect Six3 protein levels when measured at P5.5. **(B) **shRNA-mediated knockdown at P0.5 of *Six3OS *does not affect Six3 protein levels when measured at P5.5 Click here for file

Additional file 6 **Autorepression of Six3 is not affected by *Six3OS***. Transfection of CMV-*Six3 *together with the Six3-pro luciferase reporter into NIH 3T3 led to > 70% reduction in luciferase expression. Co-transfection of CMV-*Six3 *and CAG-*Six3OS *with the *Six3*-pro luciferase reporter was not significantly different than CMV-*Six3 *alone. Click here for file

Additional file 7 **List of proteins that interact with the mouse and human forms of *Six3OS *from the transcription factor/RNA binding protein microarray**. Click here for file

Additional file 8 **V5 expression constructs are expressed in HEK 293T cells**. αV5 western analysis demonstrating that Eya1-V5, Eya3-V5, Eya4-V5 and Ezh2-V5 are expressed. Click here for file

Additional file 9 **Supporting Information**. Includes plasmid information, shRNA sequences and antibodies used. Click here for file
