# Supplementary figures and images for: The long noncoding RNA Six3OS acts in trans to regulate retinal development by modulating Six3 activity
Source: Neural Dev. 2011 Sep 21;6:32. doi: 10.1186/1749-8104-6-32 (PMC3191369; doi:10.1186/1749-8104-6-32)

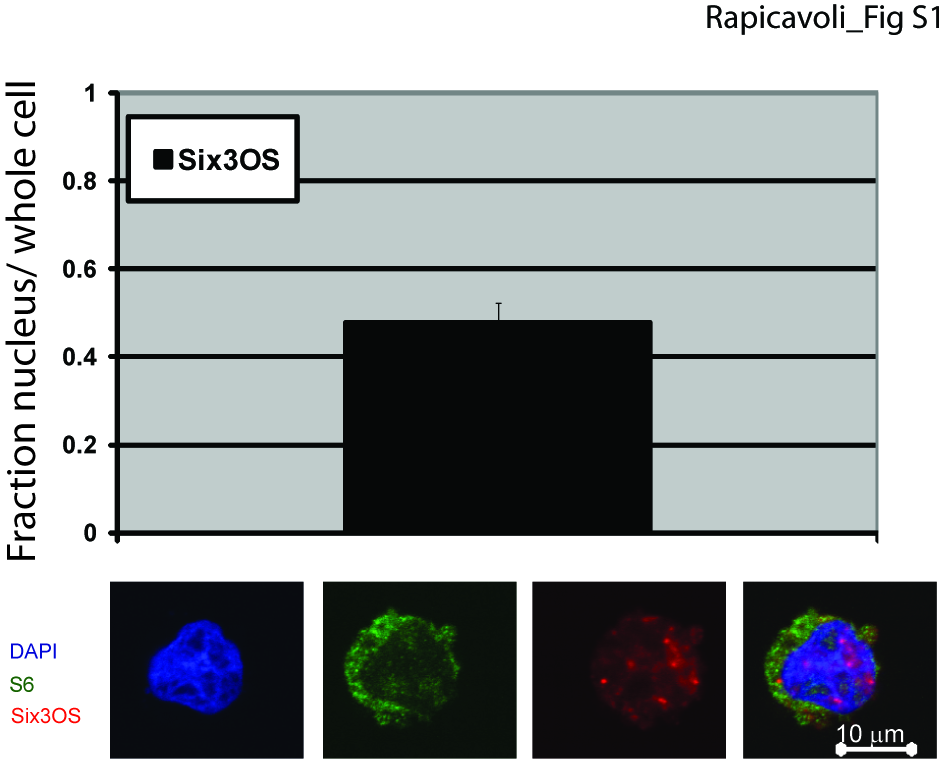

Supplement: Additional file 1 — Six3OS is localized equally in the nucleus and cytoplasm. HeLa cells were transfected with Six3OS constructs and RNA location was analyzed by FISH followed by immunohistochemistry against the cytoplasmic S6 ribosomal protein. Cytoplasmic Six3OS RNA was identified by localization with S6 protein. The relative proportion of nuclear Six3OS, defined as FISH signal that did not colocalize with S6 protein, is indicated. N = 20 cells. [file 1749-8104-6-32-S1.TIFF]
