# Supplementary material for: The long noncoding RNA Six3OS acts in trans to regulate retinal development by modulating Six3 activity
Source: Neural Dev. 2011 Sep 21;6:32. doi: 10.1186/1749-8104-6-32 (PMC3191369; doi:10.1186/1749-8104-6-32)
Supplement: Additional file 4 — Summary of overexpression and knockdown data for Six3 and Six3OS. These results demonstrate that co-expression of Six3OS and Six3 rescues the phenotypes observed with Six3 overexpression except that the photoreceptors are displaced in the outer third of the outer nuclear layer. Simultaneous knockdown of Six3OS and Six3 results in a novel phenotype, fewer amacrine cells. Additionally, Six3OS overexpression rescues the phenotype of knockdown of Six3. However, expression of Six3 combined with knockdown of Six3OS results in an additive phenotype. [file 1749-8104-6-32-S4.DOC]

**Additional File 4**

| **Gene** | **Overexpression** | **Knock down** |
| --- | --- | --- |
| **Six3OS** | **↓** syntaxin expression | **↑** Muller glia  **↓** rod bipolar cells |
| **Six3** | **↑** amacrine cells  **↓** rod bipolar cells  **↓** rod photoreceptor outer segments  **↑** rod photoreceptor cells in outer third of the outer nuclear layer | **↑** Muller glia  **↓** rod bipolar cells |
| **Six3OS &**  **Six3** | **↑** rod photoreceptor cells in outer third of the outer nuclear layer | **↓** amacrine cells |
| **↓Six3OS &**  **↑Six3** | Normal phenotype | |
| **↑Six3OS &**  **↓Six3** | **↑** Muller glia  **↓** rod bipolar cells  **↓** syntaxin expression | |
