# Supplementary material for: The long noncoding RNA Six3OS acts in trans to regulate retinal development by modulating Six3 activity
Source: Neural Dev. 2011 Sep 21;6:32. doi: 10.1186/1749-8104-6-32 (PMC3191369; doi:10.1186/1749-8104-6-32)
Supplement: Additional file 7 — List of proteins that interact with the mouse and human forms of Six3OS from the transcription factor/RNA binding protein microarray. [file 1749-8104-6-32-S7.DOC]

**Additional File 7**

| **Gene Name** | **Function** | **BC065087**  **(mouse)** | **BM663835**  **(human)** | **BX115070**  **(human** | **BM451513**  **(human)** |
| --- | --- | --- | --- | --- | --- |
| Eya1 | Transcription Factor/ Protein Phosphatase | Yes | No | No | Yes |
| Ezh2 | Chromatin Remodeling | Yes | No | No | Yes |
| SMARCE1 | Chromatin Remodeling | Yes | Yes | Yes | No |
| ENO1 | Enolase | Yes | No | Yes | Yes |
| PPP5C | Protein Phosphatase | Yes | No | No | Yes |
| KIAA0907 | Unknown | Yes | No | Yes | No |
