# Supplementary material for: The long noncoding RNA Six3OS acts in trans to regulate retinal development by modulating Six3 activity
Source: Neural Dev. 2011 Sep 21;6:32. doi: 10.1186/1749-8104-6-32 (PMC3191369; doi:10.1186/1749-8104-6-32)
Supplement: Additional file 9 — Supporting Information. Includes plasmid information, shRNA sequences and antibodies used. [file 1749-8104-6-32-S9.DOC]

**Supporting Information:**

**DNA Constructs.** pCAGIG was obtained from C. Cepko. pCAG was constructed by excising the IRES-GFP from pCAGIG with EcoRV and MscI and modifying the multiple cloning site. To make pCAG Six3OS, Six3OS was excised from BC065087 with DraI and SalI and ligated into pCAG digested with EcoRV and XhoI. To make pCAGIG Six3, pCAGIG was Gateway modified. Six3 was shuttled out of the entry clones of the Ultimate ORF Collection (Invitrogen) and into pCAGIG. pCAGIG- Gateway was modified to include a V5 tag at the N-terminus of the protein. Ezh2, Eya1, Eya2, Eya4, and Six3 were shuttled into the pCAGIG-V5 plasmid.

All shRNAs were designed with Ambion’s target finder (http://www.ambion.com/techlib/misc/siRNA_finder.html). Oligonucleotides were annealed and ligated into pSilencer 2.1–U6 hygro (Ambion, AM5760). Oligonucleotides were as follows:

Six3OS shRNA: 5’ AGCTTTTCCAAAAAAGACTTCAGTTGCCTCTCATTCTCTTGAAATGAGAGGCAACTGAAGTCG and 3’ GATCCGACTTCAGTTGCCTCTCATTTCAAGAGAATGAGAGGCAACTGAAGTCTTTTTTGGAA

Plasmid-based shRNAs directed against Six3 (clone ID TRCN0000070787) were generated by the TRC consortium and cloned into the pLKO.1 vector, expressed from the U6 promoter. pTK-Renilla was obtained from Promega. pCMV Six3 and 1.4 kb Six3 pro luciferase were obtained from Changqi Zhu (Zhu et al. 2002).

**Antibodies used.** Section immunohistochemistry: anti-glutamine synthetase (1:200, BD transduction laboratories 610518), anti-HPC1 (syntaxin) (1:200, Sigma S0664), anti-CHX10 (1:200, Exalpha Biologicals Inc X1180P) and anti-green fluorescent protein (1:1000 Invitrogen A6455). Alexa568 goat anti-mouse IgG secondary antibody (1:500 Invitrogen A11008), Alexa568 donkey anti-goat IgG secondary antibody (1:500 Invitrogen A11057), Alexa488 goat anti-rabbit IgG (1:500, Invitrogen A11004) and Alexa488 donkey anti-rabbit IgG (1:500, Invitrogen A21206).

**Dissociated retinal cell immunocytochemistry:** anti-rhodopsin (Rho4D2, 1:2000, obtained from R. Molday, University of British Columbia), anti-glutamine synthetase

(1:2000, BD transduction laboratories 610518), anti-protein kinase Cα (1:5000 Millipore 05-154), anti-HPC1 (1:2000, Sigma S0664), anti-green fluorescent protein (1:1000 Invitrogen A6455) and anti-Six3 (1:1000, abcam, ab51473). Alexa568 goat anti-mouse IgG (1:1000 Invitrogen A11008), Alexa568 goat anti-guinea pig IgG (1:1000 Invitrogen A11075) and Alexa488 goat anti-rabbit IgG (1:1000, Invitrogen A11004).

**References**

Zhu CC, Dyer MA, Uchikawa M, Kondoh H, Lagutin OV, Oliver G. 2002. Six3-mediated auto repression and eye development requires its interaction with members of the Groucho-related family of co-repressors*. Developme*n**t 1**29: 2835-2849.
